# An angle-compensating colorimetric strain sensor with wide working range and its fabrication method

**DOI:** 10.1038/s41598-022-26272-1

**Published:** 2022-12-19

**Authors:** Nguyen Hoang Minh, Kwanoh Kim, Do Hyun Kang, Yeong-Eun Yoo, Jae Sung Yoon

**Affiliations:** 1 Department of Nano Manufacturing Technology, Korea Institute of Machinery and Materials (KIMM), Daejeon, South Korea; 2 Department of Nanomechatronics, University of Science and Technology (UST), Daejeon, South Korea

**Keywords:** Mechanical engineering, Nanosensors, Sensors

## Abstract

The visual response is one of the most intuitive principles of sensors. Therefore, emission and change of the colors are widely studied for development of chemical, thermal and mechanical sensors. And it is still a challenging issue to fabricate them with a simple working mechanism, high sensitivity, good reliability, and a cost-effective fabrication process. In this study, we propose a mechanical strain sensor, which has 2D photonic crystal structures in nanoscale on stretchable polydimethylsiloxane (PDMS) substrate. Due to the periodic nanostructures, the surface of the sensor produces structural colors. And when it is stretched, the periodicity of the nanostructures changes, which results in the shift of the colors. Multiple nanostructures with different periodicities are integrated on the sensor in order to extend the working range up to 150% with high sensitivity. In addition, reusable and robust molds, which are fabricated by self-assembly of nanoparticles, are used for multiple replications of sensor substrates. Thus, the fabrication process of this study is believed to be potential for possible industrial manufacturing. This study is expected to contribute to strain sensors in the future for the applications of health care, infrastructure monitoring, soft robotics, and wearable devices.

## Introduction

In recent years, flexible and stretchable sensors have emerged as an attractive research topic due to their great potential in future applications such as smart prosthetics, e-skin, virtual reality assisted devices, human’s health monitoring^1,2^. Especially, strain sensors, which transduce mechanical deformation to readable output signal, have played a significant role in the development of flexible smart devices. To date, electric signal (capacitance or resistance) based- strain sensors have been considered as the prominent approach. While they are based on the conventional sensor principles and materials, lots of efforts have been made to enhance their stretchability. The stretchability was attained by fabricating metallic structures in nano or micro scale^3,4^. For instant, Han et al*.* deposited thin gold film on micro-cracks to mimic the vibrational sensing mechanism of the scorpion^4^. Meanwhile silver nanowires, carbon nanotubes, graphene, and mxenes were dispersed in or on the elastomeric material to achieve ultrasensitive strain sensors^5–9^. Although electric strain sensors have been well-developed, additional equipment are also required, such as electric signal processor, external power source and so on. Therefore, optical signal-based strain sensor has been suggested as an alternative approach.

Since the color is one of the fastest and most intuitive signals to recognize, it has been used for a lot of sensing mechanisms, including the mechanical strain sensor. One-dimensional photonic crystals for strain sensor have been fabricated using block copolymer^10,11^. 2D or 3D photonic crystals such as plasmonic^12^, grating ^13^, close-packed^14–16^, nonclose-packed nanoparticles (NPs)^17^, and inverse opal structure^18,19^ have been reported for strain sensors. The principle of these structures is that stretching will lead to the change of crystalline lattice, which results in the color shift of the devices. One of the technical issues of these colorimetric sensors is the angle dependency. The reflected wavelength, or color, is related to both incident and diffraction angles. So a photonic crystal sensor was fabricated to be insensitive to the viewing angle, while it had limited strain range and color intensity^20^. Lee et al. fabricated a Fabry–Perot mechanochromic interferometer with slight color shift over a board viewing angle^21^. Meanwhile, Zeng et al. embedded a fluorescent thin layer under a shielding film to demonstrate mechanochromic sensor. Although its strain range was under 60%, it could emit angle-independent colors according to the strain^22^. Another issue of the colorimetric sensors is the working range of strain. A sensor with colored hydrogel was reported by Gossweiler et al*.* by adding a bis-alkene functionalized spiropyran into PDMS. The measurable strain was up to 200%, while the color changed from purple to blue^23^. So the variation of color needs to be expanded in order to improve the sensitivity. Later on, photonic crystal hydrogels were used by Chen et al. to fabricate the strain sensor. The color shifted in the whole visible spectrum for the strain up to 570%, but it was unable to be restored to the initial shape without swelling-deswelling process^24^.

In this study, we propose a colorimetric strain sensor based on structural color, which has wide range of strain and color variation. Molds with 2D photonic crystal structures (PCS) were fabricated by self-assembly of nanoparticles, then the crystal structures were transferred onto PDMS substrate, which is the strain sensor. The color of the crystal structures changes according to the strain, but it also changes to the incident and viewing angles. So additional structures were made on non-stretchable substrate, and it was attached on the sensor substrate. Color of this substrate changes only to the incident and viewing angles, so this can be used to compensate the color change of the stretchable sensor substrate for arbitrary incident and viewing angles. The colors on the rigid substrate indicate the current incident and viewing angles, while those on the stretchable substrate indicate the color change according to the strain. Therefore, the strain could be obtained for arbitrary incident and viewing angle by comparing the colors of each substrate. And the crystal structures on the stretchable substrate were made with different periodicities, which are 780, 510 and 356 nm. Since each structure has different working ranges, the sensor substrate has wide range of measurable strain (up to 150%) with high sensitivity. And the sensor substrate showed good durability from the mechanical stretch test for over 5000 cycles. The fabrication process in this study is relatively simple and cost effective, and it is based on the robust and reusable molds. So, this study is expected to be applied for commercial strain sensors in the future.

## Method

### Preparation of molds

**Figure 1:**
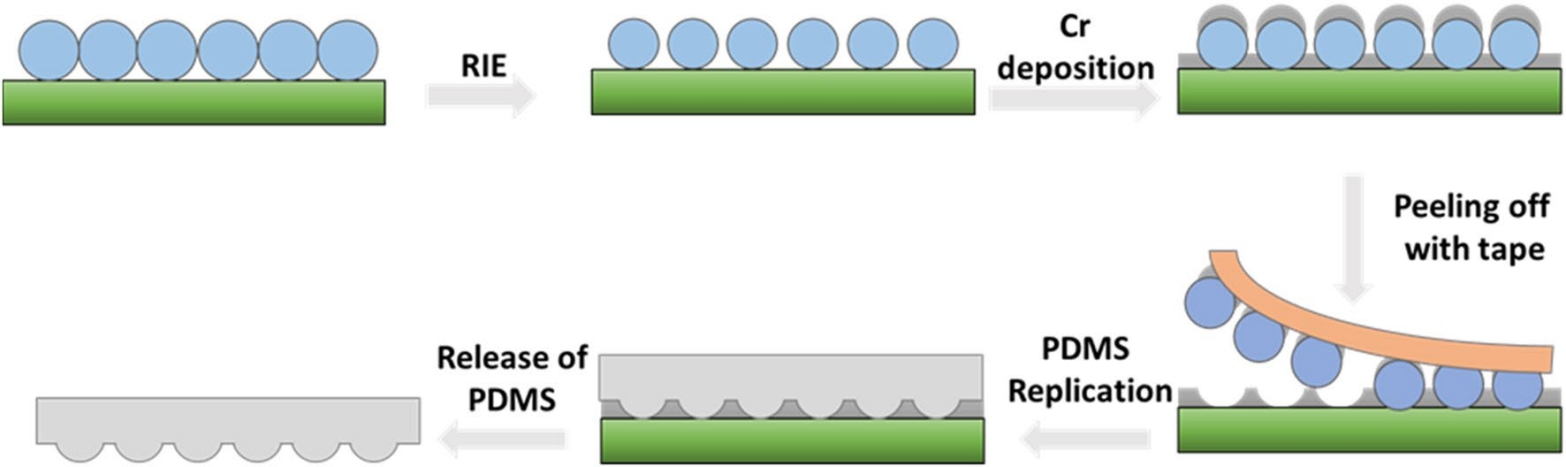
Schematic illustration of the fabrication process for mold and sensor. Nanoparticles were assembled on a silicon substrate then they were etched with RIE process. Metal (Cr) film was deposited in the gap between the particles so that concave structures were fabricated after peeling off the particles. The mold with nano-concave structures were used to fabricate sensor substrate with nano-convex structures.

**Figure 2:**
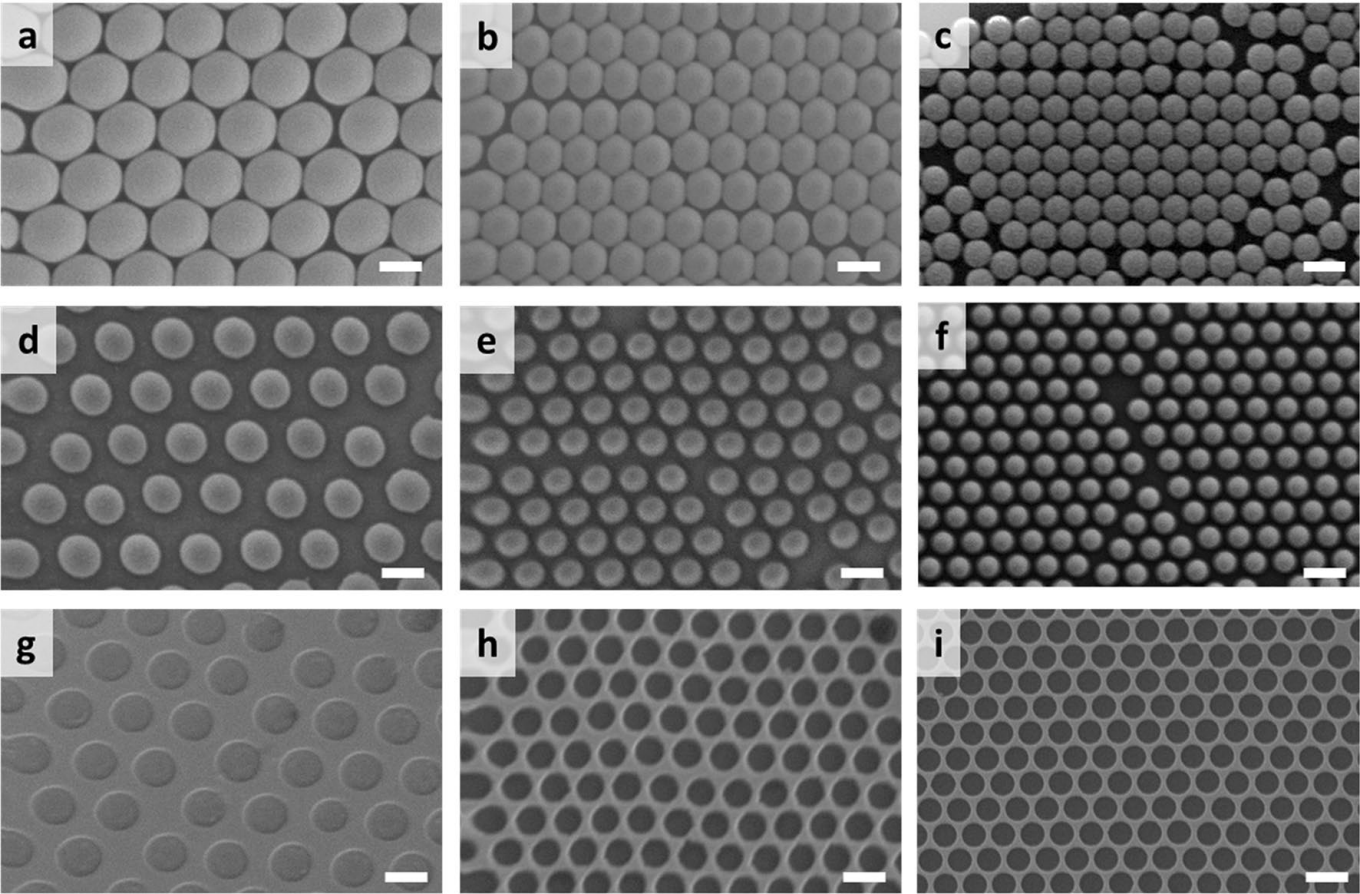
SEM images of (**a**–**f**) nanoparticles and (**g**–**i**) molds: (**a**–**c**) monolayers of (**a**) 780, (**b**) 510, (**c**) 356 nm nanoparticles. (**d**–**f**) monolayers of (**d**) 780, (**e**) 510, (**f**) 356 nm nanoparticles after RIE. (**g**–**i**) molds with periodic nanostructures from (**g**) 780, (**h**) 510, (**i**) 356 nm nanoparticles (scale bar 500 nm).

The fabrication process for the mold is illustrated in Fig. 1. The mold was made to have concave nanostructures so that the sensor substrates had convex ones after replication. We had investigated a fabrication process for nano- concave or convex structures using nanoparticles and thin film deposition^25^. Based on the fabrication method, monolayers of polystyrene nanoparticles (Bangs Laboratories, Inc.) with mean diameters of 780, 510, 356 nm were assembled on a silicon wafer by spin coating at specific areas respectively. And they were measured by a Scaning electron microscope (SEM, SM-356, Topcon) as seen in Fig. 2a–c. Next, oxygen (O_2_) reactive ion etching (RIE) was done to decrease the mean diameters of NPs (Fig. 2d–f). Then, a Cr layers with thickness of 30, 50, and 80 nm were deposited on the 780, 510, 356 nm particle areas using an e-beam evaporator, respectively. The difference of thickness of Cr layers will be explained in the result and discussion. Finally, after the removal of the particles with adhesive tape, the molds with nano-concave structures were ready for further replication process (Fig. 2g–i). We selected Cr due to its good adhesion with silicon substrate so that the metal layer could remain intact after taping and replication step.

### Preparation of strain sensor with wide working range

**Figure 3:**
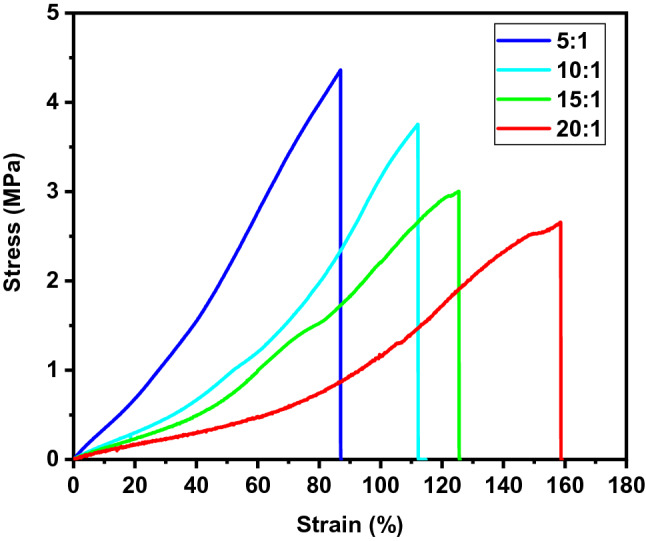
Results of tensile test with PDMS substrates for different mixing ratios (base : curing agent).

**Figure 4:**
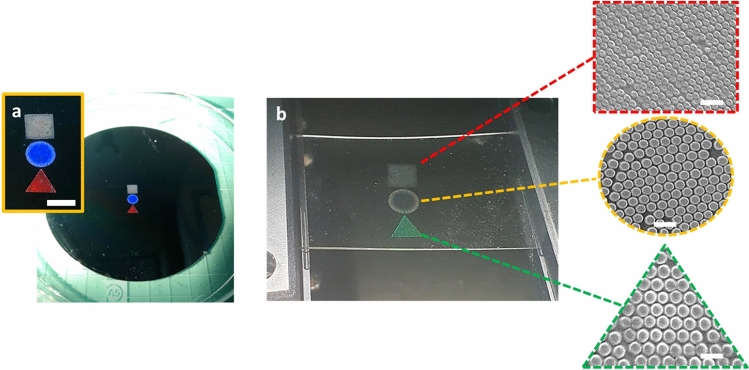
(**a**) Photo of the mold with 780, 510, and 356 nm periodic nanostructure in triangle, circle and square areas, respectively (scale bar 0.5 cm), (**b**) Photo of the sensor substrate and SEM images of 780, 510, and 356 nm periodic nanostructure in each area (scale bar 1 µm).

The replicated PDMS substrates, which are the strain sensors, need to have high stretchability. And it strongly depends on the mixing ratio of base material and curing agent of PDMS. So, a preliminary test was done with substrates of various mixing ratios. Different mixtures were prepared with ratio of 5:1, 10:1, 15:1 and 20:1 (base material: curing agent), respectively, and they were diluted with hexane at the ratio of 5:5 (wt%). The hexane was used to enhance penetration of PDMS mixture into nanostructures of the mold. The mixtures were poured onto the master mold and cured at 80 °C for 2 h. Then tensile tests were done with the substrates as seen in Fig. 3. It shows that the PDMS substrates became more ductile with decreasing curing agent content. As the result, the breaking point could reach to the strain of 160% at ratio of 20:1. In addition, The elastic modulus, calculated from the slope of the initial linear region (strain from 0 to 10%), varied from 3.3 MPa to 0.77 MPa with mixing ratio from 5:1 to 20:1, which is in accordance with the literature values^13^. Based upon this result, we fabricated the PDMS substrate with mixing ratio of 20:1 so that the sensors could tolerate the strain up to 150%. For another approach to obtain wide working range, multiple crystal structures were made with different periodicities. The nanoparticles with diameters of 780, 510 and 356 nm were used for fabrication of the mold which has separate areas for each particle as seen in Fig. 4. Different shapes in the shadow mask were used for the deposition process, so the periodicity could be distinguished by the geometric shapes, where the triangle is for 780 nm, the circle is for 510 nm and the square is for 356 nm. Figure 4 shows the nano-convex structures with periodicities of 780, 510 and 356 nm on the PDMS substrate, respectively.

## Results and discussion

### Structural color of 2D PCS and its strain-responsive characteristics

**Figure 5:**
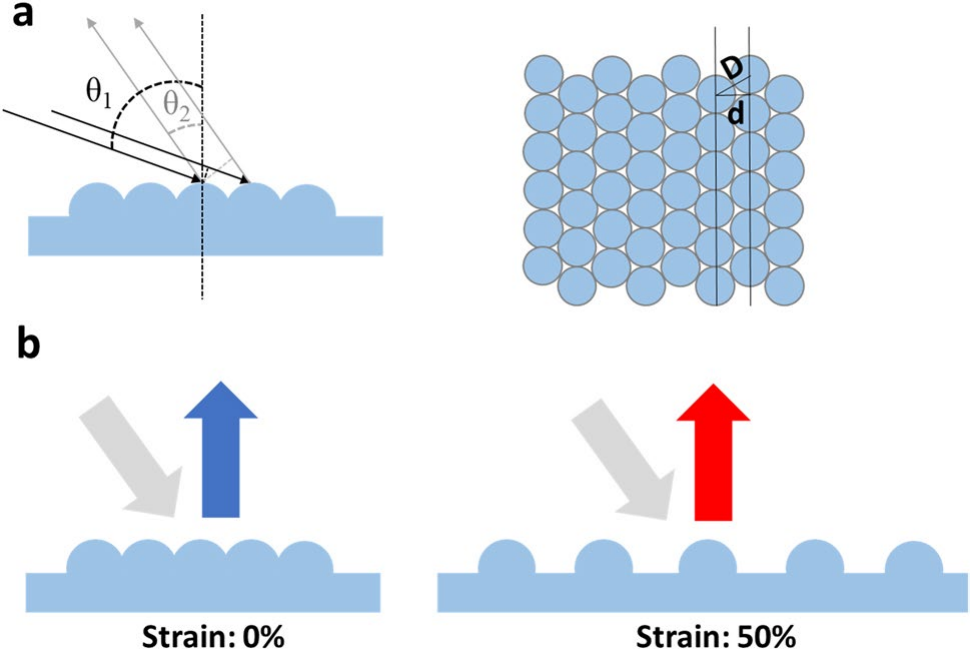
Working principle of colorimetric strain sensor based on structural color. (**a**) Schematic of the diffraction phenomenon with 2D hexagonal photonic crystal structure. (**b**) Incident light and diffracted light out of the sensor at initial (0% strain) and at stretched (50% strain) states.

There have been previous reports on the colorimetric strain sensor based on 2D PCS with both simulation and experiment^26–28^. In the literature, the PCS are in line grating or square lattice structure, where orientations of crystalline structures are uniform. However, due to the self-assembly of NPs during the spin coating step, the 2D PCS in this study has hexagonal lattices with different orientations regionally^29,30^. This phenomenon may usually occur especially when the particles are self-assembled on a large area. Therefore, we have investigated the influence of crystalline orientation on the structural color. When incident light interacts with 2D PCS at incident angle *θ*_*1*_ (Fig. 5a) and it is diffracted at angle (viewing angle) *θ*_*2*_, the difference of the light paths between 2 adjacent beams is calculated following this equation^31,32^:

1 $$\Delta = nd \, \left( {sin\,\theta_{1} + \, sin\,\theta_{2} } \right)$$

where *n* is the refractive index of external environment, which is approximate 1 for the ambient air, and *d* is the distance between adjacent lattice rows, which is equal to $\sqrt 3 /2D$(*D* is the distance between centers of two adjacent nanostructures). For the hexagonally closed-packed NPs in this study, *D* is the diameter of NP. When the light path difference is integer multiple of certain wavelength, constructive interference occurs at this wavelength as described by the following equation:

2 $$m\lambda \, = \sqrt 3 /2D \left ({ sin\,\theta_{1} + \, sin\,\theta_{2} } \right)$$

where *m* is the order of diffraction and *λ* is the diffracted wavelength. Herein, we only consider the first order of diffraction (*m* = 1). And the color appears only when the wavelength is in the invisible spectrum (400–700 nm).

**Figure 6:**
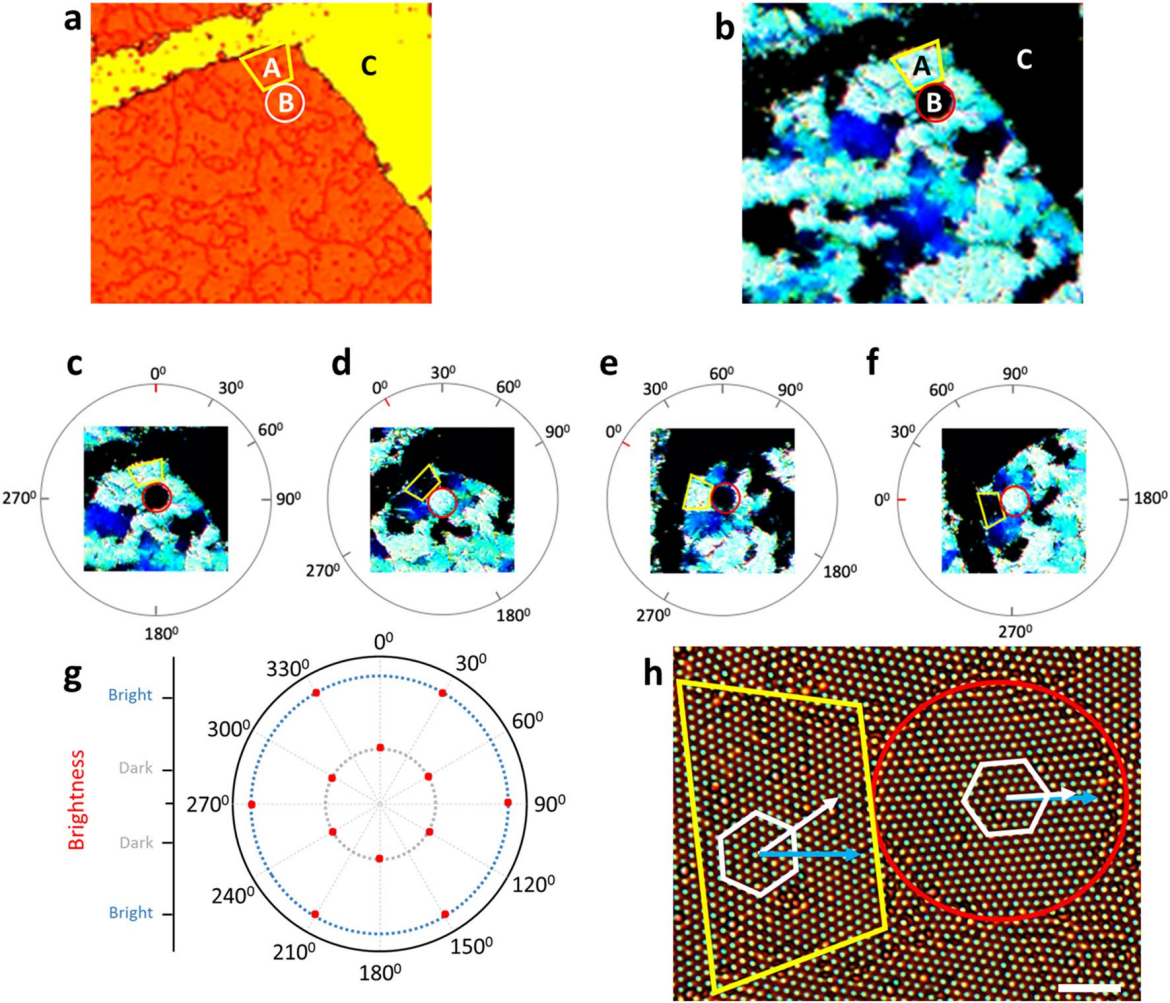
**(a**) Optical images of 2D hexagonal photonic crystal structures at reflection mode shows the areas have different brightness, (**b**) Optical images of 2D hexagonal photonic crystal structures at transmission mode shows the presence of nanoparticles in region A and B, while region C is empty. (**c–f**) Optical images of 2D hexagonal photonic crystal structures for various angles (reflection mode) show the alternating brightness of region A and B, (**g**) Polar plot of brightness of region B with rotation angle, (**h**) High magnification image (transmission mode) shows the orientations of hexagonal lattices and incident light in region A and B (scale bar 5 µm).

We observed the diffraction interaction between light and 2D PCS with hexagonal lattice using an optical microscope (OM, BX53MRF-S, Olympus) and an external halogen light source. Figure 6a is a microscopic image in transmission mode, showing the monolayer of 780 nm NPs (seen in red color) which were assembled on a glass substrate. However, when it is observed in reflection mode as seen in Fig. 6b, region A (yellow trapezoid) appeared with bright blue color, but adjacent regions B (red circle) and C were dark, even though there were particles in the region B. To explain this phenomenon, we rotated the substrate to various angles as shown in Fig. 6c–f. The brightness of 2 regions (A and B) were opposite and alternated with each other as the substrate rotated. The polar plot of brightness variation in region B is illustrated in Fig. 6g. The alternating period of the brightness is 60°, which is haft of the axial angle of 2D hexagonal lattice. And this alternation of brightness also shows that the orientation of hexagonal lattice in region A was different from that in region B for 30°. Closer observation of Fig. 6f is shown in Fig. 6h, where the orientations of those two lattices deviated by 30°. And it can be also seen that the lattice orientation of the bright region (B) was aligned with the projection of the incident light (blue arrow). Therefore, the different orientations of 2D hexagonal PCS leads to different strength of diffraction lights locally, but it does not influence the overall color when it is seen by naked eyes. Consequently, the color of the sensor substrate is made from the local regions where the lattice orientation matches that of the incident light. And when the sensor is stretched, the color changes in those bright regions while others remain dark. In detail, when the strain is *ɛ, d* shifts to *d’* = *d*_*0.*_*(1* + *ɛ)* and according to Eqs. (1) and (2), the diffracted wavelength shifts to:

3 $$\lambda^{\prime } = \, \lambda_{0} (1 + \varepsilon )$$

As shown in Eq. (3), the wavelength varies linearly with the strain. Thus, to achieve color variation over the visible spectrum from blue (450–485 nm) to red (625–750 nm), the strain range for a single sensor is determined by the ratio of those wavelengths, which is 50% (Fig. 5b).

**Figure 7:**
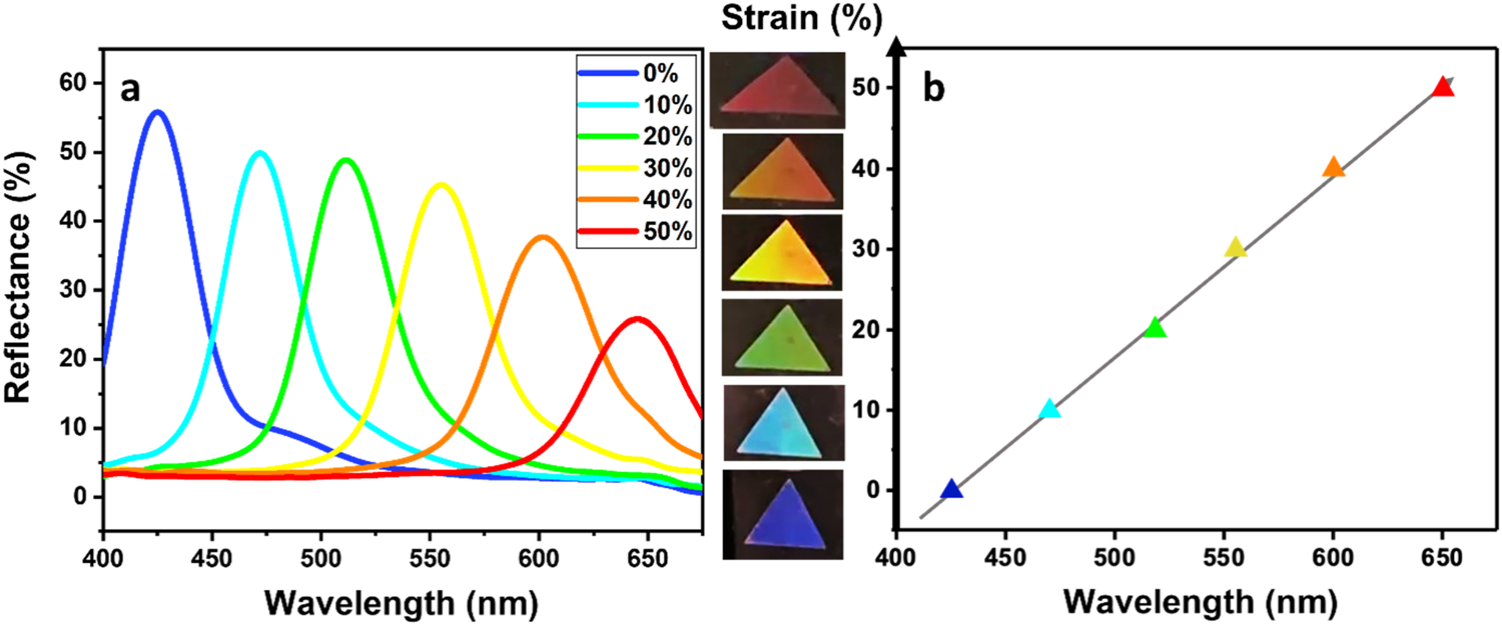
Strain-responsive characteristics of the sensor substrate which was made from 780 nm nanoparticles. (**a**) Reflectance spectra for various strain. (**b**) Colors and diffracted wavelengths for various strain and their comparison with theorical calculation (gray arrow).

The strain-responsive reflection spectra of the sensor were measured using a customized stage (Fig. S1), spectrometer (Flame-s-vis–nir, Ocean optics, Inc.), and a tungsten halogen light source. All digital photos and videos were taken with a cell phone (iPhone X) and a digital camera (EOS R5, Canon Inc.). For the 2D PCS fabricated from NPs with diameter of 780 nm, we set the incident angle *(θ*_*1*_*)* at around 40° and the camera is perpendicular to the substrate plane (*θ*_*2*_ = 0°)*.* Then the diffracted light from the sensor substrate was measured as it was stretched. The experimental results shown in Fig. 7a agree with the theoretical calculation based on Eq. (3). The wavelength shifted from 425 to 650 nm, as the strain increased from 0 to 50% (Fig. 7a). Accordingly, as the wavelength shifted linearly with applied strain, the color of sensor changed from blue (0%) to light blue (10%), green (20%), yellow (30%), orange (40%), and red (50%) (Fig. 7b). In addition, the intensity of diffracted light decreased as the strain increased (Fig. 7a). This is due to the vertical contraction of nano-convex structures as a result of substrate’s Poisson’s ratio^14,28^. Thus, for higher strain above 50%, the color returned to the initial one (blue) and changed with another cycle. But the intensity deteriorated greatly in the second cycle and afterward (Video S1), so the first cycle of the color change was considered only for the strain sensor. Furthermore, the light source and camera were parallel to the change of the periodicity, or the stretching direction of the senor, in our study. So the color of our sensor changed from blue to red as seen in the results. However, when the light source and camera were perpendicular to the stretching direction, the color changed from red to blue. It is because the periodicity for the perpendicular direction decreases due to the Poisson’s ratio^26,28^. This was also tested and shown in the supplementary information (Video S2).

### Strain sensor with angle compensator

**Figure 8:**
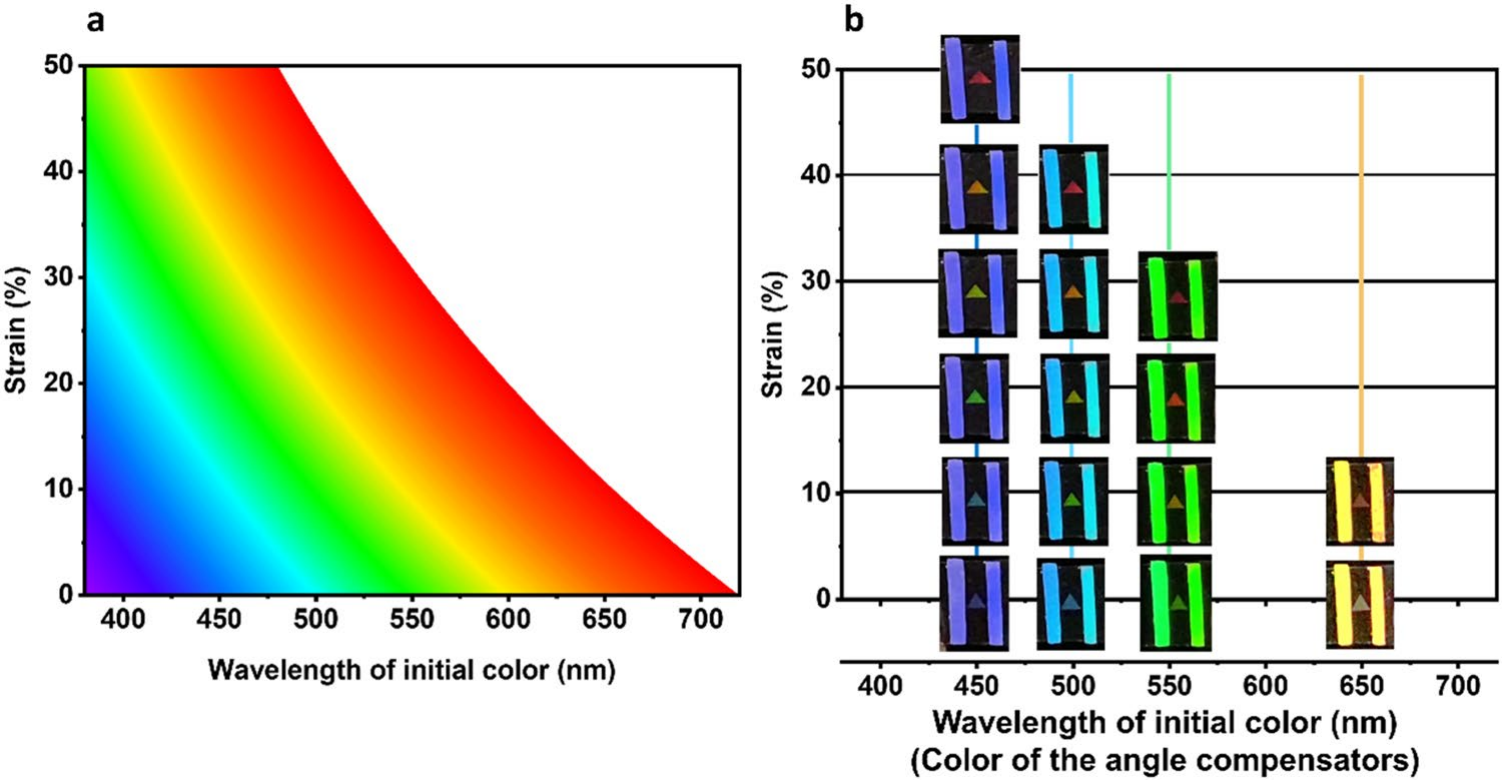
Color variation of the PCS of 780 nm with strain for different initial colors from (**a**) simulation (**b**) experiment.

Angle-dependency of the color has been a critical issue for the sensors based on PCS^10,12,18,28^. So a lot of materials and nanostructures have been studied in order to attain angle-insensitive colors^20,33–35^. The color of PCS is determined by the incident and viewing angles in Eq. (2), which is the initial color before strain is applied. Then, as the substrate is stretched, the color changes according to the strain, as seen in Eq. (3). So this variation of color can be illustrated as seen in Fig. 8a. Colors without strain (on x-axis) represent the initial ones for certain combination of incident and viewing angles (*sin θ*_*1*_ + *sin θ*_*2*_). When the strain is applied, the color of PCS changes upward in the diagram (parallel to y-axis) from the initial point. This means that the strain can be measured for any angle, as long as the initial color is known even after the sensor substrate is stretched. Therefore, additional crystal structures with periodicity of 780 nm were made on rigid silicon substrates in order to show the initial color. And they were attached on the stretchable sensor substrate as angle compensators (Fig. S2a). The angle compensators were attached on both sides of the sensor symmetrically, so that stretchable substrate could be deformed uniformly, as seen in Fig. S2b. Figure 8b shows the measuring results of the sensor substrate for different strain and angle. The stretchable sensor substrate changed its color according to the strain, while the angle compensator kept showing the initial color. Therefore, by comparing both colors, the strain could be measured regardless of the angles. Figure 8 also shows that the measuring range depends on the initial color, or the viewing and incident angles. If target range is 50% or higher as in this study, the initial color needs to be set between purple and light blue. The color of PCS of 780 nm is calculated and illustrated for various incident and viewing angles as seen in Fig. S3, which needs to be considered for setting the angles. Meanwhile, it is common in practical applications to make the incident and viewing angles the same, such as smart phones and cameras with flashlight. This is known as Littrow configuration (*θ*_*1*_ = *θ*_*2*_). The color variation for this case is shown in Fig. S4, where colors are visible for the angle from 18° to 30°, and the strain can be measured up to 50% for the angle from 18° to 21°.

### Strain sensor with wide working range

**Figure 9:**
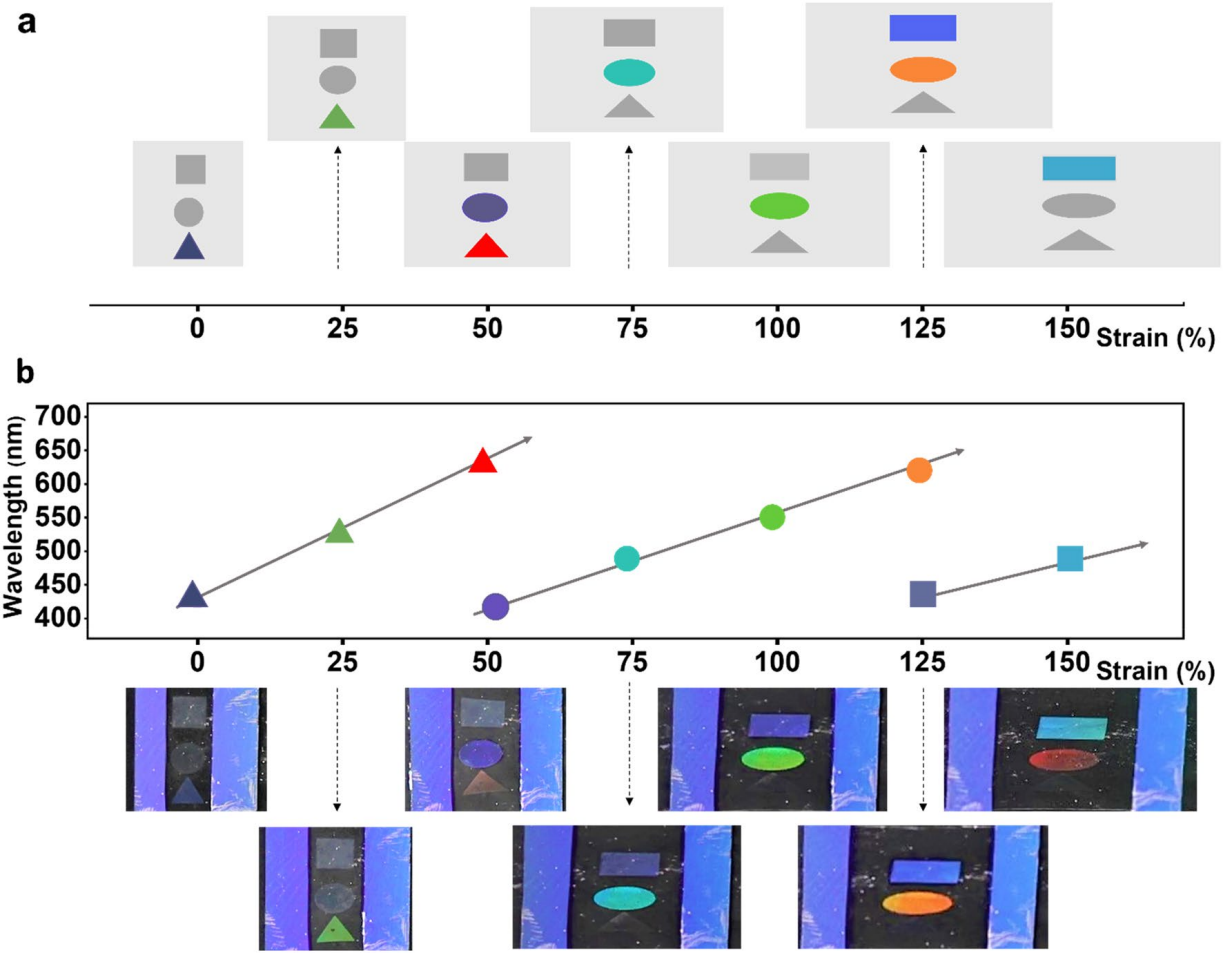
(**a**) Working principle to expand strain range up to 150% by integration of three different periodic nanostructures. 780, 510, and 356 nm periodic nanostructures were made in triangular, circular and square areas respectively. (**b**) Photos and diffracted wavelengths of triangular (0–50% strain), circular (50–125% strain), and square (125–150%) areas.

Although the sensor substrate is mechanically stretchable enough for larger strain, the variation range of visible spectrum is available only up to 50% of strain, as discussed in the previous paragraph. Therefore, we integrated three different periodic nanostructures on a single substrate so that it can measure the strain up to 150%. The working principle is schematically illustrated in Fig. 9a. The triangular area is constructed by 780 nm periodic nanostructures to measure the strain from 0 to 50%. And the circular and square areas contain 510 and 356 nm periodic nanostructures, for measuring strain from 50 to 125%, and 125% to 150%, respectively. When the sensor substrate is stretched to 50%, the periodicity in the circular area reaches 765 nm, which is similar to the initial periodicity in the triangular area (780 nm). Similarly, at 125% strain, the periodicity of nanostructure in the square area also reaches 780 nm. In addition, as aforementioned, the intensity of the color decayed as the sensor was stretched because of substrate’s Poisson’s ratio. So we fabricated those nanostructures with different heights, where higher structure was for smaller periodicity. By this way, the narrowest structures (square area) could emit vivid and visible colors even when the strain was high. As seen in Fig. 9b, when the sensor was illuminated with a white light source at initial state (strain = 0%), only the triangle appeared with blue color. From 0 to 50%, the color of triangle changed from blue to red. During further stretching the sensor from 50 to 125%, the color of triangle faded out while the circular area started shifting form blue to red. While further stretching from 125 to 150%, the square changed its color from blue to cyan. Thus, by reading the color of each area, we could evaluate the strain for the whole range wide range from 0 to 150%. It is also worthy to mention that in theory, the working range of the square could be expanded to 250% (supplementary information Table S1), however, the elongation break of PDMS in this study is under 160%, so we only considered the working range up to 150%.

**Figure 10:**
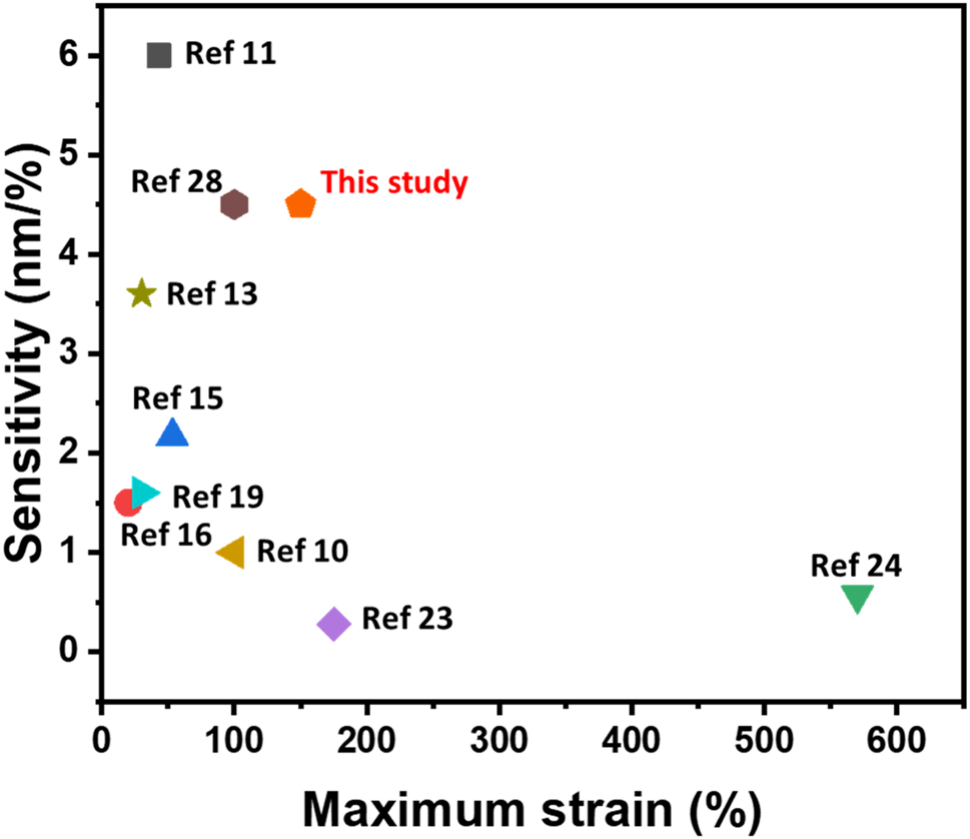
Comparison of working range and sensitivity of the stretchable colorimetric strain sensors.

In comparison with previous literature, we summarized the working range and sensitivity of various stretchable colorimetric strain sensors as shown in Fig. 10. The sensitivity is calculated by following equation:

4 $$S = \Delta \lambda /\varepsilon$$

As seen in the figure, the strain range and the sensitivity are competing parameters for the colorimetric strain sensors, as they are in general. However, the principle of this study shows quite high sensitivity and relatively wide strain range. Moreover, this study is also promising for enhancing the strain range with high sensitivity, if additional nanostructures with smaller periodicity are added.

### Sensor durability

**Figure 11:**
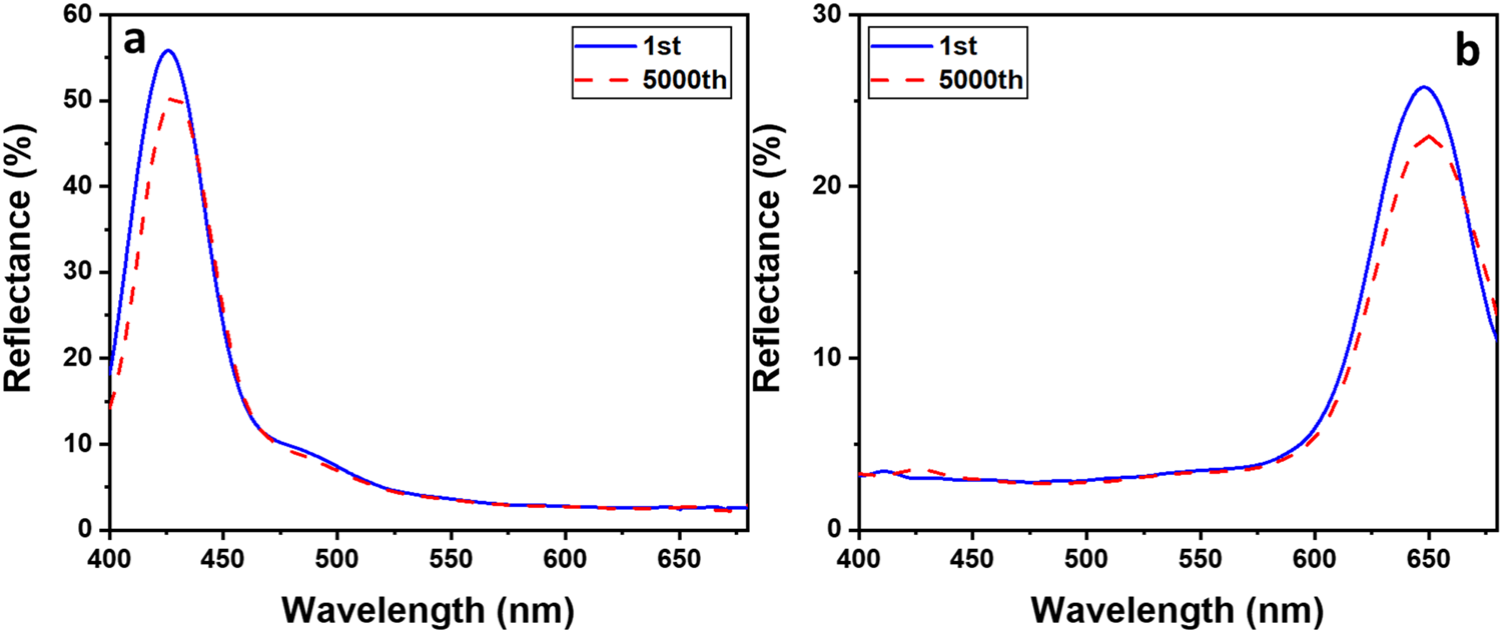
Reflectance spectra of sensor substrate after first cycle and 5000th cycle of stretching for (**a**) 0% and (**b**) 50% strain.

Since the working principle of this study is based on the mechanical stretch of the elastic substrate, its durability for restoration is crucial for practical applications. So, we performed durability test by stretching the substrate from 0 to 50% of strain for 5000 times, using a motorized stretching jig (Fig. S5). Figure 11 shows the comparison of reflection spectra before and after 5000 times of stretching, at strains of 0% and 50%. Although a slight variation occurred in the reflectance peaks, which are 2.7 nm at 0%, 1.3 nm at 50% of strain, the colors from the sensor were stable overall. This is because the material of the sensor substrate is a durable elastomer (PDMS), while other materials such as Polystyrene nanoparticles, metal (Cr) film and Si wafer are used only for fabrication of the molds. So, our principle is expected to provide higher durability for stretchable sensors.

## Conclusion

One of the major purposes of this study is to provide a simple and cost-effective process for fabrication of a strain sensor based on structural color. The sensor is simply replicated from a mold, which is fabricated by self-assembly of nanoparticles and thin film deposition. The other purpose is providing a novel method to measure the strain for various viewing and incident angles. The rigid substrate with photonic crystal structures was used as an angle compensator, which indicated the initial color for arbitrary angle. And this study also includes the investigation on the strain sensor with wide working range and high sensitivity. Strain of the sensor substrate causes change of the periodicity of the nanostructures, thus generates change of the color reflected from the sensor surface. The color changes in the whole visible spectrum from the strain of 0–50%. And the working range is expanded up to 150% by making multiple structures with various periodicities. Furthermore, the sensor substrate shows a good mechanical durability, which we believe is an advantage for future applications in the industries. As there are a lot of expectations for wearable devices, robotics, flexible sensors and so on, this study is believed to provide possible fabrication methods and sensor devices for those needs in the future.

## Supplementary Information

Supplementary Information 1.

Supplementary Video 1.

Supplementary Video 2.

## Data Availability

The datasets generated during or analysed during the current study are available from the corresponding author on reasonable request.
