# Supplementary material for: An angle-compensating colorimetric strain sensor with wide working range and its fabrication method
Source: Sci Rep. 2022 Dec 19;12:21926. doi: 10.1038/s41598-022-26272-1 (PMC9763495; doi:10.1038/s41598-022-26272-1)
Supplement: Supplementary file 1 — Supplementary Information 1. [file 41598_2022_26272_MOESM1_ESM.docx]

An Angle-Compensating Colorimetric Strain Sensor with Wide Working Range and its Fabrication Method

*Nguyen Hoang Minh ^12^, Kwanoh Kim^1^, Do Hyun Kang^1^, Yeong-Eun Yoo^12^, Jae Sung Yoon ^12*^*

^1^ Dept. Nano Manufacturing Technology, Korea Institute of Machinery and Materials (KIMM)


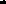


^2^ Dept. Nanomechatronics, University of Science and Technology (UST)

* [jaesyoon@kimm.re.kr](mailto:jaesyoon@kimm.re.kr)

**Supplementary Information**

**Video S1.** Strain sensor at working range from 0 to 50%

**Video S2.**  Strain sensor when the light source and camera are perpendicular to stretching direction


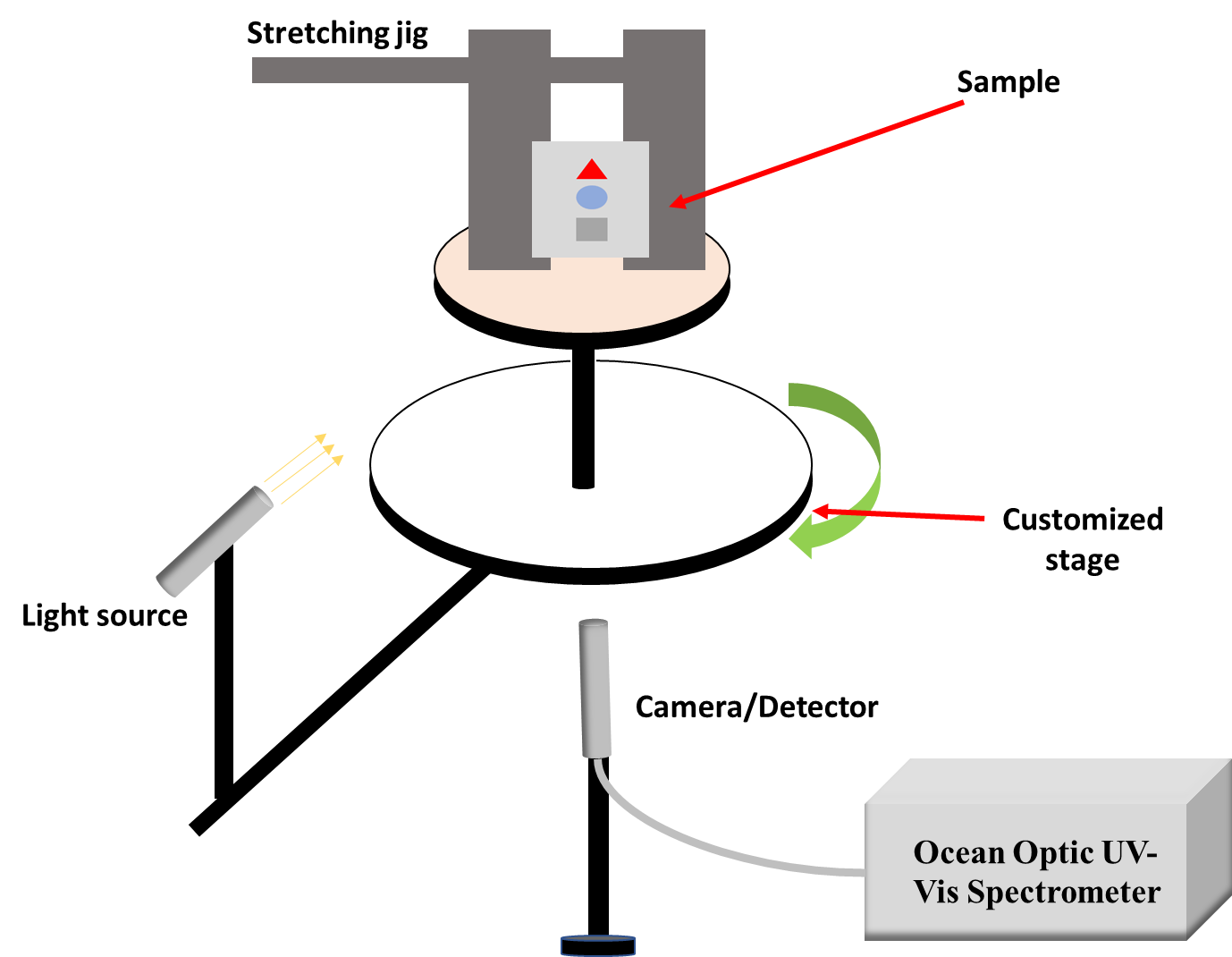


**Figure S1.** Schematic of customized stage for measuring strain-responsive spectra


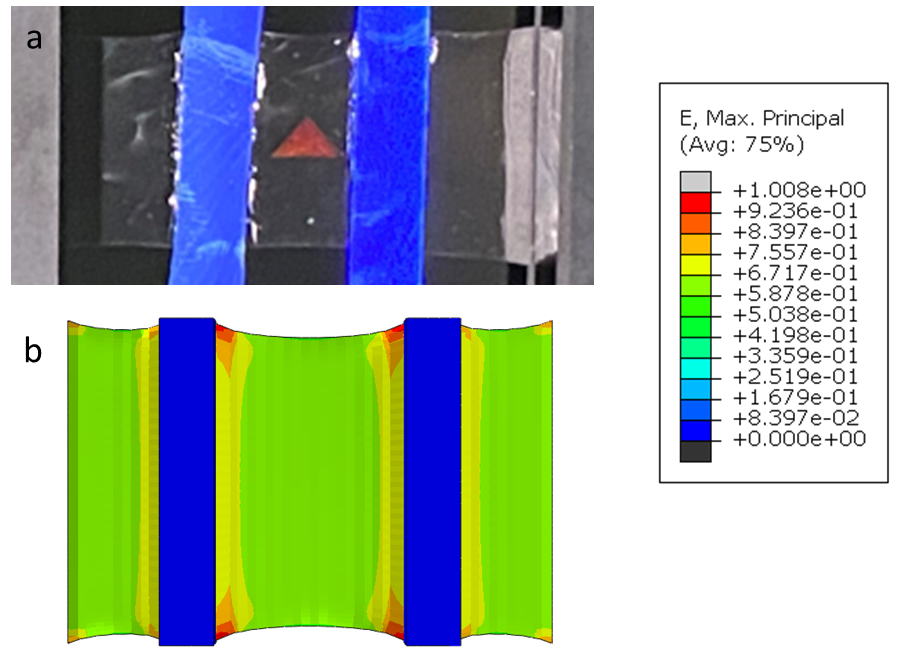


**Figure S2.** Image of the sensor with 2 rigid PCS and the simulation result of the local strain at the strain of 50%


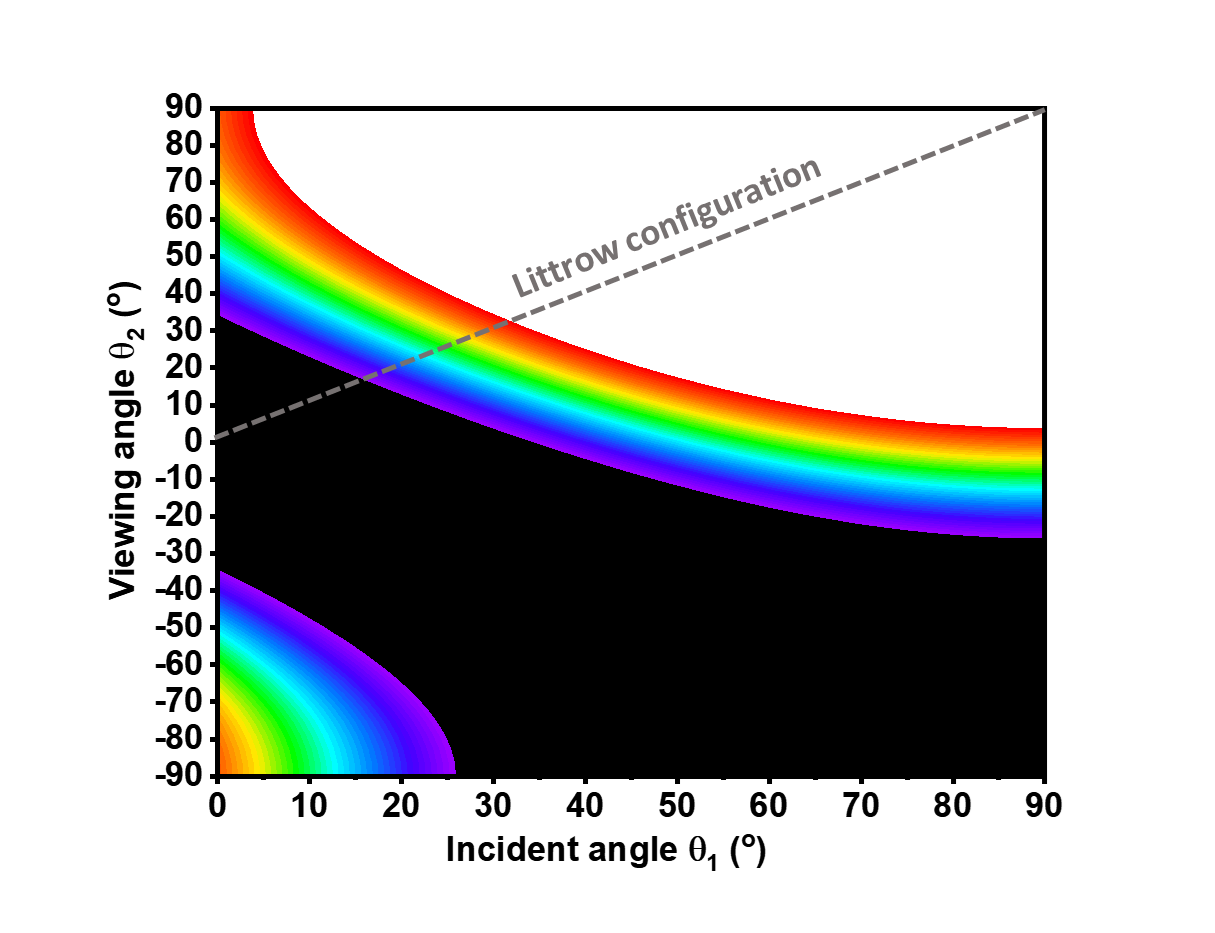


**Figure S3.** The color of 780 nm PCS at different incident and diffracted angles (black and white color mean no color is observed)


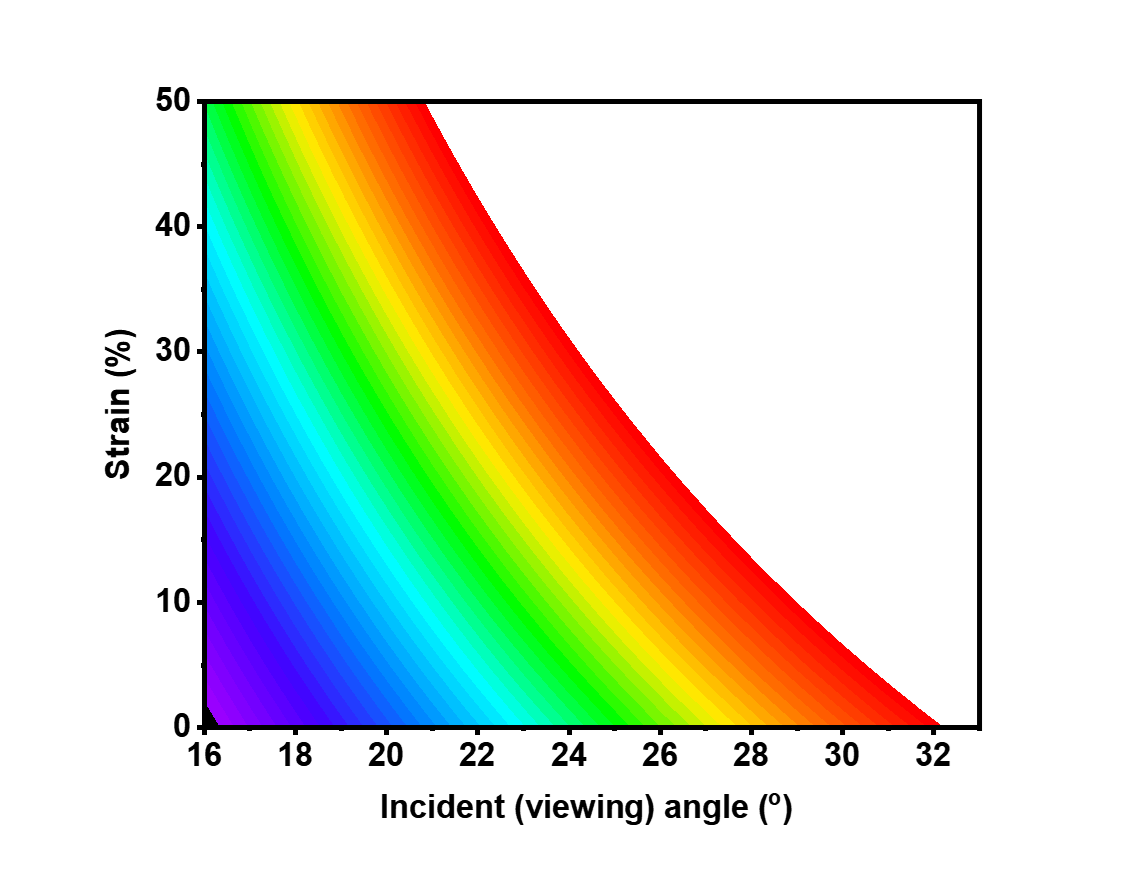


**Figure S4.** The color SPCS at different strains and viewing angles with Littrow configuration

(black and white color mean no color is observed)


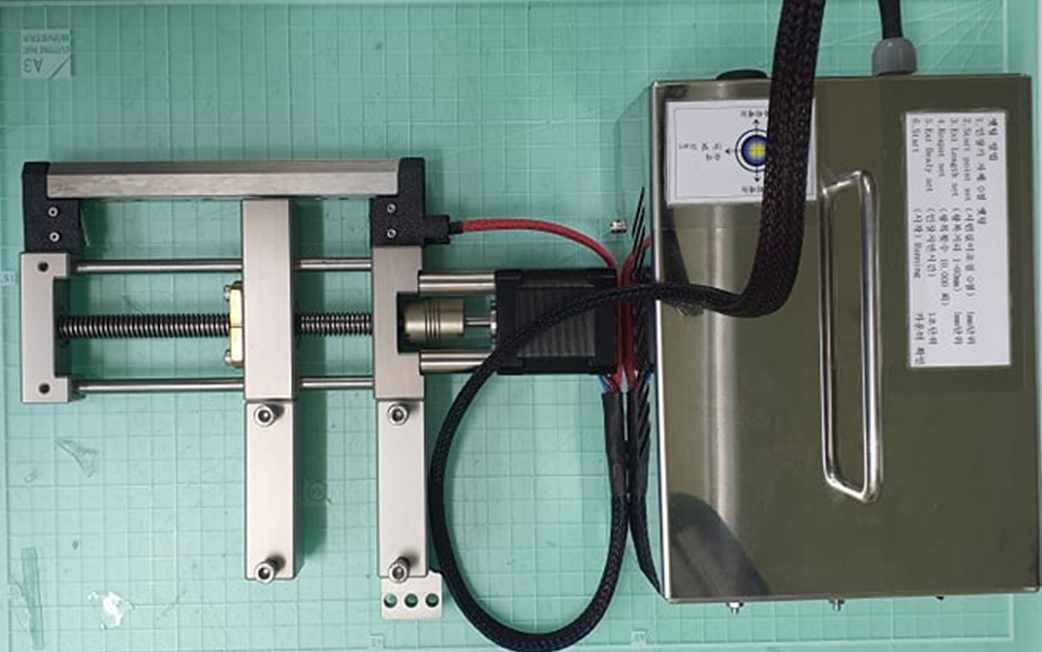


**Figure S5.** Motorized stretching jig

**Table S1.** The first order diffracted wavelengths of each pattern at different strains.

The initial diffracted wavelength of triangle is around 430 nm.

| **Strain (%)** | **Diffracted wavelength (nm)** | | |
| --- | --- | --- | --- |
|  | **Triangle** | **Circle** | **Square** |
| 0 | 430 | 281 | 196 |
| 25 | 537.5 | 351.3 | 245 |
| 50 | 645 | 421.5 | 294 |
| 75 | 752.5 | 491.8 | 343 |
| 100 | 860 | 562 | 392 |
| 125 | 967.5 | 632.3 | 441 |
| 150 | 1075 | 702.5 | 490 |
| 175 | 1182.5 | 772.8 | 539 |
| 200 | 1290 | 843 | 588 |
| 225 | 1397.5 | 913.3 | 637 |
| 250 | 1505 | 983.5 | 686 |
